# Comment on Liver Venous Deprivation or Associating Liver Partition and Portal Vein Ligation for Staged Hepatectomy? A Retrospective Multicentric Study

**DOI:** 10.1097/AS9.0000000000000125

**Published:** 2022-01-12

**Authors:** Jiali Xing, Bao Jin, Baoluhe Zhang, Shunda Du

**Affiliations:** From the Department of Liver Surgery, Peking Union Medical College Hospital (PUMCH), Peking Union Medical College (PUMC) & Chinese Academy of Medical Sciences (CAMS), Beijing, China.

## To the Editor:

We read with great interest the results of comparing liver venous deprivation (LVD) with associating liver partition and portal vein ligation for staged hepatectomy (ALPPS) for compensatory increase rates of future liver remnant (FLR). We wish to congratulate Chebaro et al^1^ for completing such a pivotal study. As indicated, this first retrospective study demonstrated a faster hypertrophy rate and higher resection rates for ALPPS than for LVD (*P* < 0.001), whereas 90-day major complication and mortality rates were comparable. However, some issues merit further discussion.

First, the authors did not explore the differences in the mechanisms of ALPPS and LVD. While comparing LVD with ALPPS in terms of the efficiency of remnant liver volume (RLV) gain, relative RLV increase was similar between LVD and ALPPS (63% vs 56%, *P* = 0.727) but with slower kinetic growth rates (KGR) in LVD than in ALPPS (2%/day vs 7%/day, *P* < 0.001). ALPPS increases FLR volume by an average of 48.6% to 90.0% in 7 to 14 days of ALPPS.^2^ Guiu et al^3^ have reported that the percentage of FLR volume increased from 28.2% to 40.9% after 21 days of LVD. ALPPS can increase active proliferation by increasing the hepatic parenchymal partition between the portal and nonportal vein embolic lobes, leading to transection of extensive communicating branches between them.^4^ Moreover, the increased levels of systemic inflammatory factors and cytokines, such as interleukin 6 and tumor necrosis factors, may have an important effect on the rapid increase in residual liver volume.^5^ LVD combines embolization of the portal inflow and venous outflow. The main mechanism explaining the effect of LVD involved the reaction of continuous miniportal inflow and a decrease in the portal collateral branches.^6^ LVD is an interventional procedure that is superior to invasive ALPPS. However, an increase in the KGR of LVD is a critical issue that needs to be solved urgently.

Second, although this study reported that the successful resection rate was significantly lower for LVD than for ALPPS (72.6% vs 90.6%, *P* < 0.001), it did not really reflect the success rate due to different indications. Therefore, it is better to explore successful resection rates aimed at primary liver tumors for LVD and ALPPS. Although ALPPS is mainly indicated for colorectal liver metastases (CRLM), it is also used for primary liver tumors. A single-center study in 2018 selected 45 patients with hepatocellular carcinoma who underwent standard ALPPS. After the first stage of ALPPS, the median increase rate of FLR reached 56.8%. A two-stage hepatectomy was completed in 41 patients (91.1%) in approximately 2 weeks. The 90-day mortality rate was 11.1%, and the 1-year and 3-year overall survival rates were 64.2% and 60.2%, respectively.^7^

Third, the authors did not report if, and for how long, they followed up these patients to evaluate the prognosis after surgery. Further follow-up (5-year survival) studies are needed to determine the effects of LVD and ALPPS, which will help determine better options in the future. Schnitzbauer et al^8^ reported that the 3-year overall survival rate of CRLM patients after ALPPS was 50%. Another study showed that the 1-, 3-, and 5-year survival rates of CRLM patients after ALPPS were 76.2%, 57.1%, and 22.9%, respectively.^9^

Finally, ALPPS did cause direct or indirect squeezing and repeated touching of the liver tumor, which may have resulted in the proliferation or metastasis of tumor cells.^10^ Postoperative liver tumor markers should be evaluated and compared between the embolized and nonembolized hepatic lobes. Moreover, the number and size of tumors should be compared before and after surgery to assess the degree of progress.

In summary, we appreciate the excellent work of Chebaro et al^1^; however, whether LVD will have a better performance is worthy of more in-depth research.
